# Protoplasmic Nutrition and Molecular Metamorphosis in the Dental Tissues

**Published:** 1887-02

**Authors:** Alton Howard Thompson

**Affiliations:** Topeka, Kansas.


AMERICAN JOURNAL

—OF—

DENTAL SCIENCE.

Vol. xx. Third Series—FEBRUARY, 1887. No. 10.

ARTICLE I.

### PROTOPLASMIC NUTRITION AND MOLECULAR METAMORPHOSIS IN THE DENTAL TISSUES.

BY ALTON HOWARD THOMPSON, D. D. S., TOPEKA, KANSAS.

[Read before the American Dental Association, August 5th, 1886 ]

All life is evolved by and in the embryonic protoplasm,—or bioplasson or sarcode,*—which, in its primitive simplicity, is the basis and matrix of all animal tissues. It is that element in all the tissues which, from its first proliferation, conveys nutrition, guides development, directs tissue-building, presides over typal evolution, and after completion of the tissue maintains the integrity of its form and quality, and carries nutriment into it and waste from it to the capillaries.

But protoplasm appears in its primitive purity in very few, if any, completed animal tissues. The complexity of organization of these various tissues is infinite, and is due to the extraneous elements, organic and inorganic, which are deposited and arranged after definite typal forms within the meshes of this primeval element of all the tissues. The embryo during its earliest stages is apparently composed of protoplasm in its simplest combinations; yet it is impressed with mysterious powers of differentiation into specified organs from the fecundated ovum, in accordance with and obedience to typal impulse. This impulse, inherent in the ovum and spermatozoa, is the great, impenetrable mystery of life. Why all the forms of all the tissues in all their multifarious qualities and compositions should assume special shapes and characteristics and powers under the impulses conveyed by the minute cells, which are the foundation of the organism—we cannot understand, unless, indeed, we fall back upon the interference of a supernatural power above and beyond our ken.

In the evolution of the organism the protoplasmic compounds, which are first proliferated as the basis structure, select from the stream of nutrient pabulum flowing through it from the capillaries the particular elements which may be necessary for elaboration of each tissue; each protoplasmic molecule of each tissue working under its own differential impulse, whether it be building bone or nerve tissue, muscle or tooth enamel. The protoplasm then remains as a permanent element of each tissue, and gives it life; it carries in the nutriment and takes out the waste. The superior, the complex elements of the tissue are therefore dependent upon the vitalizing protoplasm, not only for creation, but for subsequent support and maintenance. The complex elements make each special tissue what it is, that it may perform its allotted work in the economy of the organism. But when the vivifying protoplasm perishes, the other elements quickly die, for through it they live and move and have their being, and perform their appointed duties.

All animal tissues which lose their protoplasm through any process of evolution or exfoliation—such as the epithelial structures—become isolated and are dead They are attached to the organism, and have no life and exist unaltered if totally separated from it. It is still a mooted question whether the enamel, being an epithelial though calcified tissue, should be classified with the vital or exfoliated products. That it is totally devoid of life throughout its substance we cannot believe, nor yet that it is vital to its periphery. Perhaps a middle ground would be most nearly the truth. That it is vital is proved by the organic areas of protoplasmic elements which have been demonstrated to exist in it when the life of the pulp is maintained; but when this organ perishes the enamel becomes as dead as hair or hoof cut off from nourishing elements.

In applying these observations concerning protoplasm to a study of the teeth, we find that, like other tissues, the dentine and enamel have each been developed from a specially modified protoplasmic matrix, which in its earliest form is nearly pure albumen. With the mysterious impulse imparted in the formative pulps, they intelligently select from the pabulum the organic and inorganic elements required for the proper construction of these tissues, which are so wonderfully elaborated in all their details in accordance with typal commands.

Dentine seems to be much the same tissue as bone in regard to its chemical elements, but is very different in its morphology. It is formed in a protoplasmic matrix of excessive vascularity by the reduction of the supplied lime-salts in globules formed in successive capsules, which are pierced by the fibrils, around which permanent tubuli are formed. Spaces are sometimes isolated by the aggregation of globules, and in these there is often nothing but protoplasmic elements, but as a rule these interglobular spaces are filled by solid calcification. This form of calcific globules can be imitated experimentally by precipitating lime carbonate in any viscous fluid, but especially in fluid albumen, which comes nearest to the animal matrix. On the periphery of the dentine we find irregular granulations and spaces also, as the foundation for the more regular forms of precipitation. Throughout the whole tissue the protoplasmic elements persist as the living organic matrix which, by connection with the mains, the contents of the tubuli of the dentine, the fibrillæ, which are in their turn the persistent protoplasm, conveys nutrition into the tissue and waste from it, and incidentally conveys sensation to the pulp.

The enamel is formed in a manner analagous to that of dentine but yet dissimilar in detail, as the tissues are dissimilar in chemical composition as well as in morphology. Enamel is a calcified epithelium, or at least is formed in a protoplasmic sub-basis, which is the result of a metamorphosis of epithelium. This basis is embryonic protoplasm, and within its substance is formed the tissue which is to be the inclosing capsule of the crown of the tooth when thrust beyond the gums. The salts of lime are deposited and arranged in a specified manner, according to the transmitted impulse, and in an organic matrix of horny matter,—*keratine.* It appears that there are areas of living-matter throughout this tissue,—perhaps, indeed, a reticulum of protoplasm penetrating the substance of the prisms themselves,—but at least so far as it is living-matter it contains protoplasm, and this conveys nutrition. Through this the enamel is nourished, however limited and minute this and the corresponding removal of waste may be; the main fact being that nutrition and waste are exchanged within the tissue to some extent by means of the connection of the living-matter of the dentine with the periphery of this tissue.

The power by which the movement of fluids in the protoplasm of the tissues is maintained is the function of osmosis, inherent in the protoplasm. Upon this depends the flow of fluids which create and continue life in all animal organisms,—even that highest elaboration of protoplasm called man. For, after all, animal forms are but modified sponges, which draw fluids through the meshes of their tissues to strain out the nutritive matters contained therein and, casting their waste products into the stream, pass it onward.

It is through this faculty of osmosis inherent in all tissues that continuous currents are maintained. The well-known example of an animal diaphragm separating two dissimilar fluids which commingle more or less through this membrane, illustrates this. The membrane by a power all its own, a capillary affinity, draws the fluid within itself. That is called *end*osmosis. Then by another action—repulsion—it sends the fluid onward and outward—expels it. That is *ex*osmosis. If we now carry this conception to the living membranes and tissues within the body, we will observe that they too possess this power of osmosis; that they absorb fluids—pabulum—from the capillaries by an attraction,—an affinity,—and then expel them by a repulsion equally strong, thus maintaining the currents. The change of polarity of the fluid must take place within the tissue to account for the sudden switching from attraction to repulsion.

Probably the hunger of the tissue for food causes it to draw the pabulum, and having absorbed the nutriment it craved and thrown the waste into the current, the fluid becomes offensive, and it is expelled with equal force, thus maintaining a vacuum and acting with the precision of the positive and negative poles of the magnet. This power of attraction and repulsion must vary with the vitality and density of tissues, of course. Thus, the nerve substance being more vital and vascular would require and attract more nutriment and throw out more waste, and we find this to be a physiological fact; and bone being less vital and more dense in structure would attract less nutrition and throw out less waste. By the power of osmosis we believe that the circulation of blood and other fluids of the body is aided and accelerated, and that the attraction and repulsion so exercised on the blood by the tissues is the missing link in the chain of causes inducing the movement of the blood in its circuit.

We cannot but conclude that this function is present in all tissues; that it is one of the inherent properties of protoplasm and of all its compounds. We know, indeed, that all tissues are nourished by the circulation; we know that they throw their effete matter into the veins to be carried off; we know that all compounds of protoplasm possess the power of osmosis in both directions; we know that protoplasm is the basis of all tissue, and hence conclude that this element has most, if not all, to do with the osmotic nutrition of the tissues.

And so, as there is living-matter within the tissues of the teeth, and this living-matter must be protoplasm or its simple protean compounds, we cannot but assume that the dental tissues are nourished by the ever-present and ever-acting powers of osmosis. We know that osmosis, of course, begins at the capillary walls, the limit of the red blood-corpuscle's excursions, and that the pabulum is carried by that power to the innermost parts of the tissues, and that waste is carried back to the capillaries and there thrown into the veins, the sewage system of the economy. We know also that this osmotic circulation is maintained within the bones, by which the currents flow through the protean contents of the lacunæ and canaliculi, and the bone thereby nourished and the molecular changes effected even in calcific substance. We know also that this circulation is maintained in the fibrillæ of the dentinal tubuli, and that life is thereby sustained in the dentine. As the tubuli anastomose with the canaliculi of the cementum at the periphery of the dentine, and the circulation is continuous between the two tissues, we depend upon this circulation for the maintenance of life sufficient for the toleration of the tooth by the living tissues about it after the removal of the life-source of the dentine, the pulp. We expect it to preserve not only the life of the cementum intact, but also to maintain some vitality in the dentine in contact with it.

But further than this, it has been conclusively demonstrated that there are areas of living-matter in the enamel, and that this living-matter is in direct connection by an anastomosis more or less regular and continuous with the contents of the dentinal tubuli. If this be true, then indeed there is osmosis by which nutrition is conveyed to the enamel, however minute and inappreciable it may be.

If this circulation exists by the inherent powers of osmosis in the protoplasm or protean organic elements of the dental tissues, then we must hold that molecular change is possible within limits that could make such change appreciable. If the tissues can be fed and their waste products carried off by this osmosis, then must molecular change be possible in the dental tissues as in other tissues, through physiological variations within health-limits; but especially in favorable pathological conditions of the circulating fluids would these tissues be subject to alterations.

A few of the ordinary evidences of this alteration might be cited in illustration. First, it is well known that the teeth at eruption are not so dense in structure, so rich in inorganic elements, as at maturity. Again, this density usually increases with age and active employment, so that the dentine of old age and the dentine of adolescence are very different in quality. The former is nearly devoid of protoplasm, and the very fibrils become calcified to some extent, and often the pulp itself; while the latter, though morphologically perfect, is very incomplete chemically, and possesses a large quantity of mere protoplasm, which will need to be calcified before the dental tissues will reach their mature texture.

And if this calcification can take place after the tooth is erupted and morphologically complete, we must believe from analogy that the polarity can be reversed and *de*-calcification be possible under the incitement of pathological conditions. Even physiological change of the circulating fluids, such for instance, as occurs in pregnancy, induced perhaps by lime-starvation, may cause molecular change, for we have reasons for believing that lime is taken from the teeth and bones for the construction of the osseous system of the fœtus, and that it is returned after this function is completed. Indeed, the molecular disturbance of the entire system is very great and very appreciable, physiological activity everywhere being accelerated during the continuance of the creative function. And after this, during lactation, there is also disturbance of a somewhat different kind, a lactic prevalence and a draining of the system of its general food stores when the required lactic elements are not supplied by the digestive and assimilative powers in sufficient quantity to meet the excessive demand. In both conditions a molecular breaking down occurs, and the resulting food is appropriated by the growing child.

Again, we know that the teeth of patients which have been in good, dense condition for years, requiring very little treatment at our hands, will suddenly and often without apparent cause take on a condition of unaccountable softening, and caries will progress with uncontrollable rapidity. What it is that causes this remarkable change, what it is that acts through the circulating fluids to disintegrate and carry off the lime-salts, we do not know. But it is a molecular change of some sort,—a retrograde metamorphosis which stimulates a return to the embryonic condition,—a breaking up of molecules for *re*-formation of elements which may have a density as food to other parts of the system, or it may be to form purely waste products.

Molecular metamorphosis is at once the wonder and mystery of modern physiology. Recent investigators have completely revolutionized our ideas of even such simple things as the digestion and assimilation of foods. "The older physiologists assumed that the flesh of the meal was directly, without great effort, and without much change, so far as the chemical composition is concerned, transformed into the muscle of the eater. The researches of modern times, however, go to show that the substances taken as food undergo many changes and suffer profound disruption before they actually become part and parcel of the living body, and conversely, that the constructive powers of the animal body were grossly underrated by the earlier investigators. If we were to put forward the claim that the proteid of the meal becomes reduced almost to its elements before it undergoes synthesis into the superficially similar proteid of muscle, the energy set free in the *de*struction being utilized in the subsequent work of *con*struction, we would not anticipate modern research but for a brief time, for it would almost seem as if the qualities of each particle of living protoplasm were of such individual character that it had to be built up fresh almost from the very beginning. The problems of physiology in the future will be largely concerned in arriving, by experiment and inference,—by the mind's eye and not by the body's eye alone,—at a knowledge of the molecular construction of this protean protoplasm, of the laws according to which it is built up, and those by which it breaks down; for these laws when ascertained will clear up the mysteries of the protean work which the protoplasm does. All over the body the protoplasm is constantly building itself up out of the pabulum supplied by food, and continually breaking down, giving rise to different tissues and combinations in different parts of the body, with different compositions and different properties, the various activities of the body being the outcome of the various properties of the various combinations. If this be true, it inevitably follows that protoplasm cannot be the same everywhere, but that there must be many varieties of protoplasm with different qualities, and with correspondingly different molecular structure and composition."—*Michael Foster.*

But as to this molecular metamorphosis in the substance of the tissues,—both constructive and destructive, progressive and retrogressive,—we cannot yet witness its methods, but only its phenomena. "We are taught by physiologists that there is a constant splitting up of the molecules of which the body is composed. This breaking up is accompanied in health by corresponding building up of tissues from the food after assimilation. For instance, the bones lose their phosphate of lime to which they owe their solidity, the salt passes into the blood and is there eliminated by the kidneys. The brain also loses phosphorous. Now, the food should, if appropriated and duly assimilated, supply an abundance of the phosphates,—ample, indeed, to bear the strain imposed upon the phosphorous resources of the body. But this supply being insufficient, or digestion or assimilation being imperfect as regards the phosphorous salts, a phosphorous famine begins and the body feeds upon itself and consumes its own phosphorous. So a general malnutrition may react upon any specified parts of the organism by abducting its constituent salts, albumen or other matters, and thereby lessen its resistive power to disease; and the teeth are organs peculiarly liable to suffer from this general malnutrition, inducing a chronic starvation, and by lowering their vitality and robbing them of much of this reserve store of materials render them more liable to caries."—Ed. *British Journal of Dental Science.*

Molecular metamorphosis is the cause of every act and process of nutrition and removal of waste. Indeed, it is certain that a " wasting of its tissues " *is* wasting,—a removal of integral parts by molecular breaking down, but the expected replacement does not follow. In certain diseases assimilation and molecular construction seem to be held in abeyance, while breaking down and waste still go on, either normal or abnormal, and in other conditions again waste is lessened and construction goes on, with corresponding increase in the quantity or density of the tissue. These operations we observe in the dentine, and perhaps they take place in the enamel. As there is nutrition and waste by osmetic currents in these tissues, there must be molecular change in accordance with general law. The teeth cannot be exceptions to a rule among vital tissues in this regard. These can be waste of the inorganic elements of the dentine and probably of the enamel, and when this removal is not followed by compensating reconstruction of lime phosphate molecules, softening of these tissues results, with corresponding lessening of resistance to the attacks of caries. Then, again, there may be increase of density, as transpires with age, by a more rapid construction than waste. If molecular progression and retrogression are continuous in normality, then is metamorphosis omnipresent in the dental as in all other tissues.

ADDENDA.

In the condition known as inflammation in the dentine, we have a molecular activity which seems to be a breaking down and removal of lime-salts, then of its organic matrix, and then in healing a reconstruction of both occurs. "Inflammation causes solution of the lime-salts, and afterwards a liquefaction of basis-substance, both in bone and dentinal substance. The result will be the appearance of globular spaces, a bay-like excavation, which, instead of being filled with basis-substance, exhibits medullary corpuscles multinuclear protoplasmic masses, corresponding to the embryonal stage of the tissue . . . Suppuration may result, but far more common is the healing process of eburnitis, the results of which may be seen in the formation of dentine closely resembling secondary dentine, or a dentine destitute of canaliculi,—osteo-dentine."—*Heitzmann and Bodecker.* What this inflammatory condition is we will not stop to consider, but it will suffice for our purpose to direct attention to the intense molecular activity that takes place, the breaking down and then total removal first of organic and then of inorganic elements, and then the following of molecular synthesis, by which the basis-substance, as well as the calcified elements, is reproduced again. Here is a molecular work which cannot for a moment be doubted, and, while we observe its operations with wonder, we cannot but regret that the mystery of its *modus operandi* is yet impenetrable.—*Dental Cosmos.*

